# The role of inflammation in takotsubo syndrome: A new therapeutic target?

**DOI:** 10.1111/jcmm.18503

**Published:** 2024-06-19

**Authors:** Xiao Li, Jingmin Jing Yang, Danyan Xu

**Affiliations:** ^1^ Department of Internal Cardiovascular Medicine Second Xiangya Hospital, Central South University Changsha Hunan China

**Keywords:** cytokines, immune therapy, macrophages, neutrophils, takotsubo syndrome

## Abstract

Takotsubo syndrome (TTS) is a particular form of acute heart failure that can be challenging to distinguish from acute coronary syndrome at presentation. TTS was previously considered a benign self‐limiting condition, but it is now known to be associated with substantial short‐ and long‐term morbidity and mortality. Because of the poor understanding of its underlying pathophysiology, there are few evidence‐based interventions to treat TTS. The hypotheses formulated so far can be grouped into endogenous adrenergic surge, psychological stress or preexisting psychiatric illness, coronary vasospasm with microvascular dysfunction, metabolic and energetic alterations, and inflammatory mechanisms. Current evidence demonstrates that the infiltration of immune cells such as macrophages and neutrophils play a pivotal role in TTS. At baseline, resident macrophages were the dominant subset in cardiac macrophages, however, it underwent a shift from resident macrophages to monocyte‐derived infiltrating macrophages in TTS. Depletion of macrophages and monocytes in mice strongly protected them from isoprenaline‐induced cardiac dysfunction. It is probable that immune cells, especially macrophages, may be new targets for the treatment of TTS.

## 1 INTRODUCTION

Takotsubo syndrome (TTS) was first reported by Sato et al. in 1990.^1^ Patients with TTS classically present with acute‐onset chest pain, dyspnea, and changes on ECG after sudden unexpected stress and major physical illness or trauma.^2^ Typical coronary angiography of TTS usually shows nonobstructive coronary arteries with a characteristic anteroseptal‐apical dyskinetic ballooning of the left ventricle.^3^

With the rising prevalence of modern life stressors and the greater awareness and detection of the condition by clinicians, the incidence of TTS is increasing.^4^, ^5^ TTS accounts for approximately 2%–3% of all patients and 5%–6% of female patients presenting with suspected acute myocardial infarction.^2^, ^5^, ^6^, ^7^, ^8^, ^9^ Approximately 90% of TTS patients are female; compared with female patients, male patients are younger, have a higher prevalence of comorbid conditions, and have higher rates of cardiogenic shock and in‐hospital mortality.^10^ Approximately 90% of patients with TTS were ≥50 years of age, but younger patients with TTS had a numerically higher in‐hospital mortality than middle‐aged and elderly TTS patients.^11^ TTS was previously considered a benign self‐limiting disease, but it is now known to be associated with substantial short‐ and long‐term morbidity and mortality.^5^, ^12^, ^13^, ^14^, ^15^, ^16^, ^17^

Despite being described three‐decades ago, the pathogenesis and pathophysiology of TTS remain poorly understood. To date, the mainstream opinion is that the pathogenesis and pathophysiological mechanisms involved in TTS include endogenous adrenergic surge,^18^, ^19^, ^20^, ^21^ psychological stress or preexisting psychiatric illness,^19^, ^22^, ^23^, ^24^ coronary vasospasm with microvascular dysfunction,^25^, ^26^, ^27^, ^28^ metabolic and energetic alterations,^29^, ^30^, ^31^ and inflammatory mechanisms. In recent years, growing evidence has demonstrated that immune cell infiltration plays an important role in TTS. Therefore, we focused on the role of immune cell infiltration in TTS in this review, and exploring a new target for the treatment of TTS.

## 2 MACROPHAGES INFILTRATION IN TTS

### 2.1 Clinical reserch

Macrophages are perhaps the most studied immune cell infiltrates in the pathogenesis of TTS. In 2007, Nef et al. discovered macrophages (CD68) in the acute phase of TTS via immunohistochemical staining, which were also detected in the recovered phase of TTS.^32^ In another multicenter study, Scally et al. recruited 55 patients with TTS and 51 matched control subjects and detected inflammatory macrophages in the myocardium via ultrasmall superparamagnetic particles of iron oxide (USPIO) enhancement during the acute phase of TTS and at the 5‐month follow‐up. They demonstrated that there was an increase in myocardial macrophages in patients with acute TTS compared with control subjects. At 5 months, inflammatory macrophages were no longer detectable in the left ventricular myocardium.^29^

In a multicenter study, although there was no difference in the total monocyte count during the acute phase, patients with TTS had a higher percentage of classic CD14^++^CD16^−^ monocytes, a lower percentage of intermediate CD14^++^CD16^+^ monocytes, and a lower percentage of non‐classic CD14^+^CD16^++^ monocytes than control subjects.^29^ The percentages of classic (CD14^++^CD16^−^) and non‐classic (CD14^+^CD16^++^) subpopulations became comparable to those of control subjects after 5 months, whereas the intermediate subset (CD14^++^CD16^+^) remained suppressed. This suggests that the increased percentage of classic monocytes is the result of an immediate release of classic monocytes from the bone marrow (and spleen) into the circulation or the infiltration of intermediate and non‐classic monocytes into myocardial tissue. It is likely that the phagocytosing macrophage infiltrate detected in the myocardium originates from the migration of these circulating monocytes into the heart, rather than the proliferation of resident myocardial macrophages.

### 2.2 Basic reserch

As in the clinical studies described above, similar results were found in rodents. In an experimental rat model of TTS induced by a single dose of isoprenaline intraperitoneally, Wilson et al. detected that macrophage counts peaked at Day 5 following isoprenaline injection and were typically found in regional clusters via immunohistochemical analysis.^33^ Macrophage infiltrates subsided by Day 5 but had not returned to baseline levels at Day 14.^33^ Furthermore, the authors examined the inflammatory phenotype of macrophages and found that proinflammatory M1 macrophages increased over time after injection, peaked at Day 4, decreased between Day 4 and Day 5 and then peaked a second time from Day 6 to Day 14.^33^ However, the anti‐inflammatory M2 macrophages showed a completely different pattern; they increased from Day 0 to Day 1, and did not change significantly from Day 1 to Day 6, but fell on Day 7.^33^

Liao et al. established a mouse model of TTS by administering a single dose of isoprenaline in intraperitoneally, sorted CD45^+^ cells from mouse hearts 24 h after injection, and performed single‐cell RNA‐Seq studies. They identified 10 distinct cell populations, the macrophage cluster being the most abundant in the TTS group at approximately 2‐fold the level of the control group.^34^ In addition, they investigated the source of macrophages in the heart. At baseline, Tim4^+^ resident macrophages were the dominant subset. In response to isoprenaline stimulation, the cardiac macrophage subsets underwent a shift from Tim4^+^ resident macrophages to Tim4^−^ infiltrating macrophages, suggesting that monocyte infiltration was the dominant mechanism of macrophage expansion in the TTS heart.^34^

To summarize these studies, a normal heart has its own tissue‐resident cardiac macrophages that display an M2‐like phenotype, which produce anti‐inflammatory, proangiogenic and pro‐reparative factors. Following isoprenaline injection, M1 macrophages or monocyte‐derived macrophages, which are associated with tissue damage, come to dominate and their inflammatory actions can unleash functions that promote disease.^33^, ^34^, ^35^, ^36^ Therefore, could targeting M1 macrophages or monocyte‐derived macrophages benefit patients by promoting myocardial repair and functional improvement in TTS?

### 2.3 Macrophage, a new therapeutic target?

**FIGURE 1:**
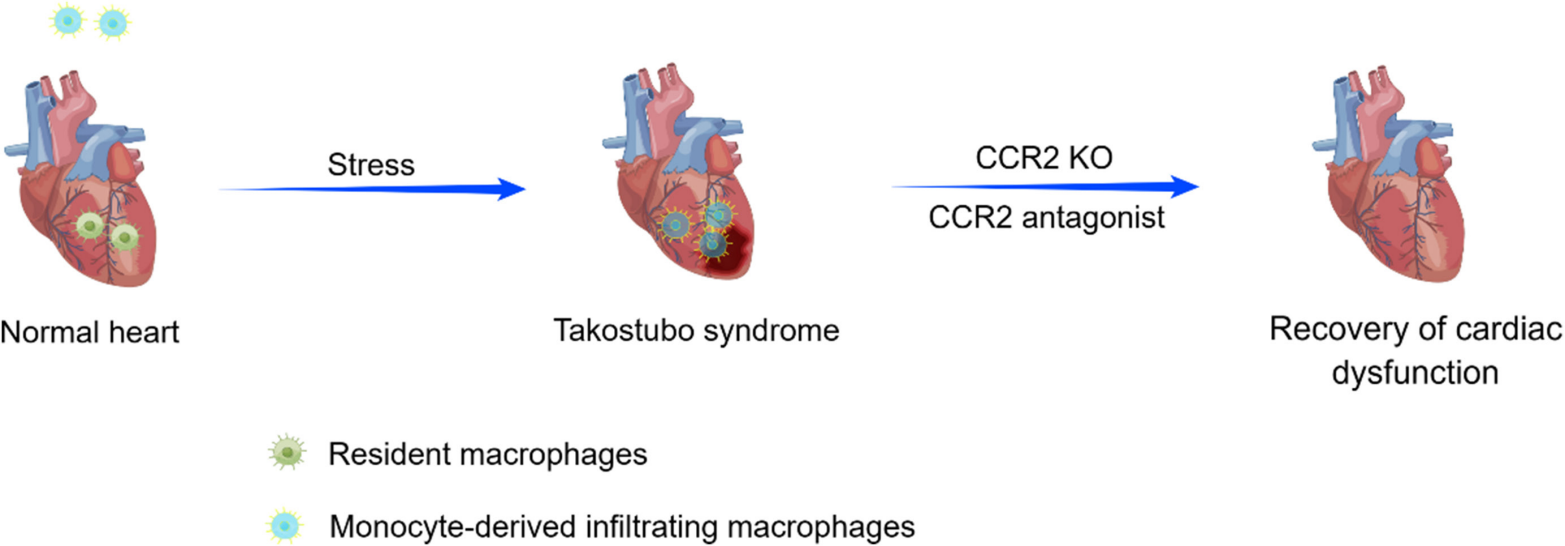
Monocyte‐derived infiltrating macrophages as a critical regulator of TTS pathogenesis. In normal heart, resident macrophages were the dominant subset. In response to stress, the cardiac macrophage subsets underwent a shift from resident macrophages to monocyte‐derived infiltrating macrophages. Blockade of macrophage infiltration (via a CCR2 antagonist or in CCR2‐KO mice) resulted in recovery of cardiac dysfunction in TTS mice.

To verify this hypothesis, Liao et al. found that mice pretreated with clodronate‐containing liposomes (depleted of macrophages and monocytes) exhibited profound protective resistance to isoprenaline‐induced cardiac dysfunction.^34^, ^37^, ^38^ Moreover, they found that compared with WT mice, CCR2‐KO mice, which lacked circulating monocytes and were detective in chemotaxis, had significantly preserved left ventricular ejection fraction (LVEF) after isoprenaline injection.^34^, ^37^ Then they treated WT mice with RS‐504393, a small‐molecule CCR antagonist, and found that RS‐504393 administration significantly improved LVEF.^34^ These results suggest that M1 macrophages or monocyte‐derived macrophages not only play critical roles in TTS pathogenesis but also serve as targets for the development of novel TTS therapies (Figure 1).

## 3 OTHER IMMUNE CELLS IN TTS

### 3.1 Neutrophils

In a rat model of TTS, Wilson et al. found that early neutrophilic infiltrates predominated at 6 h following isoprenaline injection, and peaked at Day 1 to Day 2.^33^ This is a very different pattern from that of macrophages (Table 1), suggesting that neutrophils may be the earliest immune cells infiltrating the myocardium in TTS.

**TABLE 1:** Characterization of immune cell infiltration in TTS.

Immune cell	Characterization
M1 macrophages	Increased over time, peaking at Day 4, decreased between Day 4 and Day 5 and then peaked a second time from Day 6 to Day 14
M2 macrophages	Increased from Day 0 to Day 1, did not change significantly from Day 1 to Day 6, fell on Day 7
Neutrophils	Increased 6 hour, peaking at Day 1to Day 2, subsiding at Day 3

In human studies, there is evidence of myocardial neutrophil infiltration and elevated circulatory neutrophil counts in patients with TTS. Iio et al. performed a myocardial biopsy on the day of admission of a 29‐year‐old woman with TTS and found neutrophilic infiltration in the myocardium.^39^ Similar results were seen in several other autopsy cases.^40^, ^41^ In a histopathologic study, the myocardium of 25 patients who died of subarachnoid haemorrhage and 18 controls was stained with antibodies identifying neutrophil granulocytes (myeloperoxidase). The findings revealed that in the myocardium of subarachnoid haemorrhage patients, the number of myeloperoxidase‐positive cells was significantly higher than it was in controls.^42^

Circulatory neutrophil counts were elevated in the early stages of TTS and were associated with poor prognosis. Yan et al. analysed nine patients with TTS and found that five of nine patients had an increased percentage of neutrophils.^43^ In a multicenter prospective registry study, 160 consecutive patients with TTS were enrolled to evaluate whether the assessment of the neutrophil/lymphocyte ratio (NLR) at admission could be useful to predict clinical outcomes during hospitalization of TTS patients. The results demonstrated that NLR at admission was significantly higher in patients with in‐hospital complications (IHC), and it predicted IHC, even in multivariate analysis.^44^ In addition to IHC, the NLR was associated with overall mortality and long‐term outcome. Ahn et al. included 231 patients identified with TTS and retrospectively reviewed IHC and overall mortality and found that a higher NLR was associated with a higher risk of IHC and overall mortality.^45^ In another multicenter retrospective analysis that included 338 patients with TTS, high NLR was a significant predictor of severe IHC, and the highest NLR tertile was significantly associated with lower 5‐year survival. These studies suggested NLR is a new easy‐to‐measure tool to predict worse short‐ and long‐term outcome after TTS.^46^

Activation of neutrophils is a critically important component of the innate immune, system and culminates in the ‘neutrophil burst’, which facilitates neutrophil phagocytosis via the release of superoxide anion radical (O_2_^−^) from NADPH oxidase.^47^, ^48^, ^49^ Excessive and/or prolonged neutrophil activation results in substantial tissue injury and increases vascular permeability, resulting in sustained tissue infiltration with neutrophils and monocytes, and persistent vasomotor dysfunction.^50^ Such changes were seen in TTS. B‐type natriuretic peptide (BNP), acting via inhibition of activation of neutrophil NADPH oxidase, is an important negative modulator of the “neutrophil burst”.^51^ Under TTS, BNP loses virtually all its ability to suppress neutrophil O_2_^−^ release and its impact on tissue inflammation. In addition, BNP responses do not recover for at least 3 months after an attack, suggesting that this might contribute to the perpetuation of myocardial inflammation in TTS patients.^52^

### 3.2 T cells

There have been few reports on T cells in TTS. Nef et al. studied eight patients presenting with TTS, and serial myocardial biopsies were taken during the phase of severely impaired left ventricular function and after functional recovery.^32^ They detected an increased number of T cells (CD3) besides macrophages (CD68) in the acute phase in comparison to the recovered phase via immunohistochemistry.^32^ Their findings suggested that T cells may be involved in the pathophysiological process of the acute phase of TTS.

TTS is closely associated with neurological stimulation, such as in epilepsy. Ji et al. performed correlation analysis of immune cells with co‐expressed genes for CIBERSORT and rank sum tests between immune cells in an epilepsy dataset, and performed the same operation on a TTS dataset.^53^ The results showed that the overall immune infiltration differed between epilepsy and TTS, with a higher proportion of neutrophils in epilepsy. In contrast, the proportion of T cells was greater in TTS, and some genes, such as the LEP gene, which were moderately correlated with T cells, were also increased in TTS.^53^ These results suggested that myocardium obtained T cell‐related genes in TTS, rather not simply T cells infiltration in the acute phrase of TTS, inflammatory mechanisms do not play a transient role in TTS as previously described.

These results suggested that in addition to macrophages, T cells may also play an important role in TTS. However, there were no more studies focus on T cells in TTS. Future studies such as targeting T cells in TTS will further confirm the role of T cells in TTS.

## 4 CYTOKINES IN TTS

Immune cells secrete cytokines to promote or suppress inflammation. Cytokines involved in TTS include IL‐1β, IL‐2, IL‐4, IL‐6, IL‐10, TNF‐α and IFN‐γ. Santoro et al. analysed circulating levels of cytokines in 32 TTS patients and 32 acute coronary syndrome (ACS) patients at admission and at Day 5 after admission.^54^ At admission, patients with TTS presented higher serum levels of IL‐2, IL‐4, IL‐10, TNF‐α and IFN‐γ but a lower level of IL‐6 than those with ACS. At Day 5, patients with TTS compared to those with ACS presented higher serum levels of IL‐2, IL‐1β and IFN‐γ, and a lower level of IL‐6.^54^ These results suggested that different inflammatory patterns can be observed during the acute and subacute phases of TTC when compared to ACS. During the acute phase of TTC, serum levels of anti‐inflammatory cytokines (IL‐2, IL‐4 and IL‐10) are higher than those of ACS patients. During the subacute phase of TTC, anti‐inflammatory cytokines still have higher concentrations than they do in ACS patients. In another study, Scally et al. evaluated serum cytokines in 55 TTS patients and 51 control subjects at admission and at the 5‐month follow‐up.^29^ They found that patients with acute TTS had higher serum concentrations of IL‐6 than control subjects. Although the concentrations of IL‐6 fell at follow‐up compared with the initial presentation, they remained elevated compared with those of the control subjects.^29^ The difference between the two studies in the variation trend of IL‐6 may be due to their control groups: one was ACS patients, and the other was age‐, sex‐ and comorbidity‐matched subjects. After all, patients with ACS show higher levels of IL‐6 than healthy controls.

In addition, cytokines may be predictors of outcome in patients with TTS. A prospective study enrolled 56 consecutive subjects with TTS and evaluated circulating levels of IL‐6 and IL‐10 at admission and at a mean of 178 days of follow‐up.^55^ They found that increased serum levels of IL‐6 and IL‐10 were associated with higher adverse event rates at follow‐up and higher mortality rates.^55^

Unfortunately, even though such studies have demonstrated that cytokines are increased in TTS, there have been no further studies that tested interventions against these cytokines for the treatment of TTS. This may be the investigate direction for the new therapy target in TTS.

## 5 FUTURE DIRECTIONS

Coronary angiography is the gold standard for diagnosing TTS, but it is an invasive operation. There is an urgent need for more pertinent cardiac biomarkers, ideally to diagnose TTS rapidly and noninvasively. Cytokines secreted from immune cells and conveniently detected in blood are associated with TTS, such as IL‐6. Unfortunately, although there are slight differences in some cytokine levels between ACS and TTS, they are not sufficient to distinguish TTS from ACS. In addition, the cause‐effect relationship between cytokines and TTS remains unknown. In recent years, new TTS markers have been emerging. A clinical study by Solberg et al.^56^ enrolled 20 TTS patients, found that compared with control group, soluble T‐cell immunoglobulin mucin domain‐3 (sTIM‐3) was increased in TTS patients. sTIM‐3 is thought to reflect monocyte/macrophage activation, suggested that sTIM‐3 as a potential novel marker to diagnose TTS.

**FIGURE 2:**
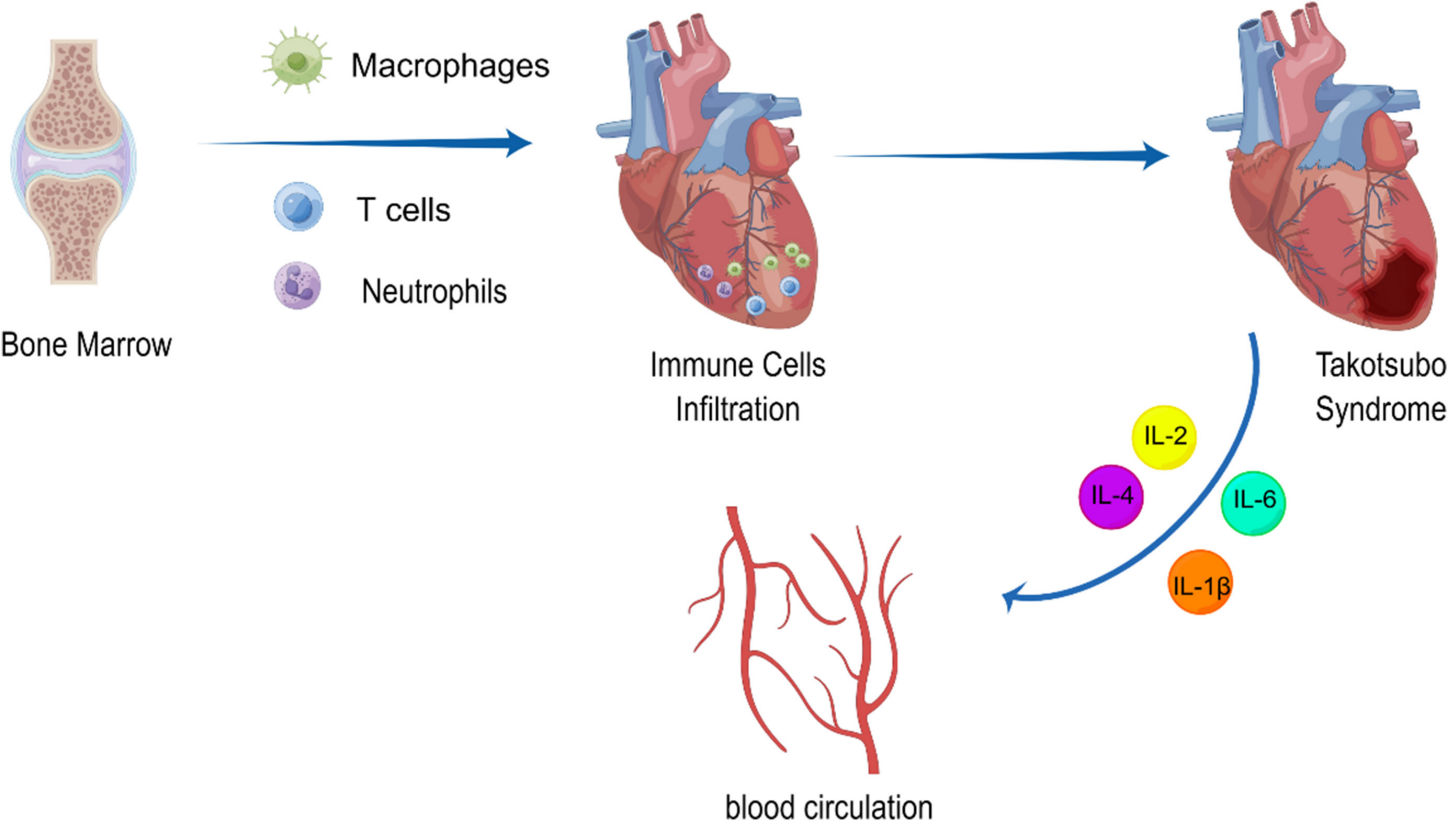
The role of immune cell infiltration in Takotsubo syndrome. Bone marrow‐derived macrophages, T cells and neutrophils migrate to and infiltrate in the heart, in the presence of other pathogenic factors, leading to TTS. These immune cells secrete various cytokines such as IL‐2, IL‐6, etc., and enter the circulation with the blood.

There is a major lack of evidence to guide management in patients with TTS, and it is essential to continue exploring the mechanism of TTS. So far, endogenous adrenergic surge, psychological stress or preexisting psychiatric illness, coronary vasospasm with microvascular dysfunction, metabolic and energetic alterations and inflammatory mechanisms have been put forth to explain TTS. It is clear that immune cell infiltration, particularly macrophage infiltration, is an integral part of the disease (Figure 2), even most studies have merely expounded on the correlation between TTS and immune cell infiltration. However, one study verified macrophage infiltration as the cause of TTS, by demonstrating that depletion of macrophages and monocytes led profound protective resistance to isoprenaline‐induced cardiac dysfunction. This is a great advancement even though it was only in animals, and we can expect macrophages to be a new target for the treatment of TTS. However, other immune cells that have been shown to be associated with TTS, such as neutrophils or T cells, have not been confirmed whether targeting with them improves TTS. Future studies need to focus on whether these cells have the potential to be targets for treatment of TTS.

In addition, TTS has been treated with steroids or immunosuppressants in some case reports, suggested that use of steroids or immunosuppressants to suppress systemic inflammation to treat TTS seems to be an option. However, the adverse effects of cortisol or immunosuppressants should not be ignored. Therefore, biologics or monoclonal antibodies that target specific immune cell types or cytokines involved in TTS are important directions in the future. Furthermore, new technologies such as longitudinal single‐cell sequencing analysis or other high‐resolution immunoassays are used to comprehensively test the role of immune cells in TTS to deepen our understanding of the role of inflammation in this disease, as well as to develop more rational, mechanism‐based treatments.

## AUTHOR CONTRIBUTIONS

**Xiao Li:** Conceptualization (equal); data curation (equal); formal analysis (equal); investigation (lead); methodology (lead); project administration (lead); resources (lead); software (lead); supervision (lead); validation (lead); visualization (lead); writing – original draft (lead); writing – review and editing (equal). **Jingmin Jing Yang:** Writing – review and editing (equal). **Danyan Xu:** Conceptualization (equal); funding acquisition (lead).

## FUNDING INFORMATION

This work was supported by National Natural Science Foundational of China (No. 81871858 and No. 82172550).

## CONFLICT OF INTEREST STATEMENT

The authors declared that they have no conflicts of interest to this work.

## CONSENT FOR PUBLICATION

All authors approved the final manuscript and the submission to this journal.

## Data Availability

Data sharing not applicable to this article as no datasets were generated or analysed during the current study.
